# Surgical Treatment of Postinfarction Ventricular Septal Rupture

**DOI:** 10.1001/jamanetworkopen.2021.28309

**Published:** 2021-10-20

**Authors:** Daniele Ronco, Matteo Matteucci, Mariusz Kowalewski, Michele De Bonis, Francesco Formica, Federica Jiritano, Dario Fina, Thierry Folliguet, Nikolaos Bonaros, Claudio Francesco Russo, Sandro Sponga, Igor Vendramin, Carlo De Vincentiis, Marco Ranucci, Piotr Suwalski, Giosuè Falcetta, Theodor Fischlein, Giovanni Troise, Emmanuel Villa, Guglielmo Actis Dato, Massimiliano Carrozzini, Giuseppe Filiberto Serraino, Shabir Hussain Shah, Roberto Scrofani, Antonio Fiore, Jurij Matija Kalisnik, Stefano D’Alessandro, Vittoria Lodo, Adam R. Kowalówka, Marek A. Deja, Salman Almobayedh, Giulio Massimi, Matthias Thielmann, Bart Meyns, Fareed A. Khouqeer, Nawwar Al-Attar, Matteo Pozzi, Jean-François Obadia, Udo Boeken, Nikolaos Kalampokas, Carlo Fino, Caterina Simon, Shiho Naito, Cesare Beghi, Roberto Lorusso

**Affiliations:** 1 Department of Cardiothoracic Surgery, Heart and Vascular Centre, Maastricht University Medical Centre, Maastricht, the Netherlands; 2 Department of Medicine and Surgery, Circolo Hospital, University of Insubria, Varese, Italy; 3 Clinical Department of Cardiac Surgery, Central Clinical Hospital of the Ministry of Interior in Warsaw, Warsaw, Poland; 4 Cardiothoracic Surgery Department, San Raffaele University Hospital, Milan, Italy; 5 Department of Medicine and Surgery, Cardiac Surgery Clinic, San Gerardo Hospital, University of Milano-Bicocca, Monza, Italy; 6 Department of Medicine and Surgery, University of Parma, Cardiac Surgery Unit, University Hospital of Parma, Parma, Italy; 7 Department of Experimental and Clinical Medicine, “Magna Graecia” University of Catanzaro, Catanzaro, Italy; 8 Department of Cardiovascular Anesthesia and Intensive Care, Istituto di Ricovero e Cura a Carattere Scientifico (IRCCS) Policlinico San Donato, San Donato Milanese, Italy; 9 Department of Cardio-Thoracic Surgery, University Hospital Henri-Mondor, Assistance Publique–Hopitaux de Paris Créteil, Paris, France; 10 Department of Cardiac Surgery, Medical University of Innsbruck, Innsbruck, Austria; 11 Cardiac Surgery Unit, Cardio-Thoraco-Vascular Department, Niguarda Hospital, Milan, Italy; 12 Cardiothoracic Department, University Hospital of Udine, Udine, Italy; 13 Cardiac Surgery Unit, IRCCS Policlinico San Donato, San Donato Milanese, Italy; 14 Section of Cardiac Surgery, University Hospital, Pisa, Italy; 15 Department of Cardiac Surgery, Cardiovascular Center, Klinikum Nürnberg, Paracelsus Medical University, Nuremberg, Germany; 16 Cardiac Surgery Unit, Poliambulanza Foundation Hospital, Brescia, Italy; 17 Cardiac Surgery Department, Mauriziano Hospital, Turin, Italy; 18 Cardiovascular and Thoracic Surgery Department, King Fahad Medical City, Riyadh, Saudi Arabia; 19 Cardiac Surgery Unit, Luigi Sacco Hospital, Milan, Italy; 20 Department of Cardiac Surgery, Medical University of Silesia, School of Medicine in Katowice, Katowice, Poland; 21 Department of Cardiac Surgery, Upper-Silesian Heart Center, Katowice, Poland; 22 Department of Thoracic and Cardiovascular Surgery, West-German Heart Center, University of Duisburg-Essen, Essen, Germany; 23 Department of Cardiac Surgery, University Hospitals Leuven, Leuven, Belgium; 24 Department of Cardiac Surgery, King Faisal Specialist Hospital and Research Center, Riyadh, Saudi Arabia; 25 Department of Cardiothoracic Surgery, Golden Jubilee National Hospital, Glasgow, Scotland; 26 Department of Cardiac Surgery, Louis Pradel Cardiologic Hospital, Lyon, France; 27 Department of Cardiovascular Surgery, University Hospital Düsseldorf, Heinrich Heine University, Düsseldorf, Germany; 28 Cardiovascular and Transplant Department, Papa Giovanni XXIII Hospital, Bergamo, Italy; 29 Department of Cardiovascular Surgery, University Heart & Vascular Center Hamburg, Hamburg, Germany; 30 Cardiovascular Research Institute Maastricht, Maastricht, the Netherlands

## Abstract

**Importance:**

Ventricular septal rupture (VSR) is a rare but life-threatening mechanical complication of acute myocardial infarction associated with high mortality despite prompt treatment. Surgery represents the standard of care; however, only small single-center series or national registries are usually available in literature, whereas international multicenter investigations have been poorly carried out, therefore limiting the evidence on this topic.

**Objectives:**

To assess the clinical characteristics and early outcomes for patients who received surgery for postinfarction VSR and to identify factors independently associated with mortality.

**Design, Setting, and Participants:**

The Mechanical Complications of Acute Myocardial Infarction: an International Multicenter Cohort (CAUTION) Study is a retrospective multicenter international cohort study that includes patients who were treated surgically for mechanical complications of acute myocardial infarction. The study was conducted from January 2001 to December 2019 at 26 different centers worldwide among 475 consecutive patients who underwent surgery for postinfarction VSR.

**Exposures:**

Surgical treatment of postinfarction VSR, independent of the technique, alone or combined with other procedures (eg, coronary artery bypass grafting).

**Main Outcomes and Measures:**

The primary outcome was early mortality; secondary outcomes were postoperative complications.

**Results:**

Of the 475 patients included in the study, 290 (61.1%) were men, with a mean (SD) age of 68.5 (10.1) years. Cardiogenic shock was present in 213 patients (44.8%). Emergent or salvage surgery was performed in 212 cases (44.6%). The early mortality rate was 40.4% (192 patients), and it did not improve during the nearly 20 years considered for the study (median [IQR] yearly mortality, 41.7% [32.6%-50.0%]). Low cardiac output syndrome and multiorgan failure were the most common causes of death (low cardiac output syndrome, 70 [36.5%]; multiorgan failure, 53 [27.6%]). Recurrent VSR occurred in 59 participants (12.4%) but was not associated with mortality. Cardiogenic shock (survived: 95 [33.6%]; died, 118 [61.5%]; *P* < .001) and early surgery (time to surgery ≥7 days, survived: 105 [57.4%]; died, 47 [35.1%]; *P* < .001) were associated with lower survival. At multivariate analysis, older age (odds ratio [OR], 1.05; 95% CI, 1.02-1.08; *P* = .001), preoperative cardiac arrest (OR, 2.71; 95% CI, 1.18-6.27; *P* = .02) and percutaneous revascularization (OR, 1.63; 95% CI, 1.003-2.65; *P* = .048), and postoperative need for intra-aortic balloon pump (OR, 2.98; 95% CI, 1.46-6.09; *P* = .003) and extracorporeal membrane oxygenation (OR, 3.19; 95% CI, 1.30-7.38; *P* = .01) were independently associated with mortality.

**Conclusions and Relevance:**

In this study, surgical repair of postinfarction VSR was associated with a high risk of early mortality; this risk has remained unchanged during the last 2 decades. Delayed surgery seemed associated with better survival. Age, preoperative cardiac arrest and percutaneous revascularization, and postoperative need for intra-aortic balloon pump and extracorporeal membrane oxygenation were independently associated with early mortality. Further prospective studies addressing preoperative and perioperative patient management are warranted to hopefully improve the currently suboptimal outcome.

## Key Points

**Question:**

What are the early outcomes of surgical treatment of postinfarction ventricular septal rupture?

**Findings:**

In this cohort study of 475 patients from 26 different centers worldwide, the early mortality rate for surgically treated ventricular septal rupture was 40.4%, mostly due to low cardiac output, and it did not improve in the last 2 decades.

**Meaning:**

The findings of this study suggest that patient-tailored preoperative and perioperative management of postinfarction ventricular septal rupture should be addressed to improve the current suboptimal survival rates.

## Teaser

This cohort study assesses the clinical characteristics and early outcomes for patients who received surgery for postinfarction ventricular septal rupture and identifies factors independently associated with mortality.

## Introduction

Ventricular septal rupture (VSR) is a rare but life-threatening mechanical complication of acute myocardial infarction (AMI).^1,2^ Due to the introduction and continuous improvement of early percutaneous revascularization strategies, its incidence has decreased to approximately 0.25% of AMI cases.^3,4,5,6^ However, if left untreated, it is almost inevitably fatal.^2^ Nevertheless, even when prompt surgery can be offered, in-hospital mortality remains very high. The rate of in-hospital mortality has not changed over the years, making VSR the most lethal cardiac surgical condition.^1,3,6^

Percutaneous closure devices and mechanical circulatory supports (MCS) may offer alternative or synergistic strategies to treat these patients, both preioperatively and postoperatively.^7,8^ However, surgery remains the standard treatment for postinfarction VSR. Early repair is generally recommended and is often required because of the presence of hemodynamic instability, but clear indications on ideal management and timing of intervention have not yet been well established.^3,6,9,10^ Moreover, given the low incidence of this condition, most reports consist of single-center studies with limited patient cohorts or studies of national registries.^1,11,12,13,14,15,16^

We conducted an international, multicenter, retrospective study on mechanical complications of AMI, the Mechanical Complications of Acute Myocardial Infarction: an International Multicenter Cohort (CAUTION) Study. The investigation was designed to evaluate early outcomes and to identify possible prognostic factors associated with early mortality among patients who underwent surgical repair of postinfarction VSR.

## Methods

### Patient Population and Study Design

The patients were recruited from the database of the CAUTION study (NCT03848429), a retrospective multicenter trial aimed at evaluating the postoperative outcomes of patients undergoing surgery for post-AMI mechanical complications. The study described in this article included all adult patients (aged >18 years) who underwent surgical repair of postinfarction VSR between January 2001 and December 2019 in 26 different centers worldwide, identified through surgical records. The study protocol was authorized by the local ethical committees of each center and conducted in accordance with the guidelines of the Declaration of Helsinki^17^ for patient data use and evaluation. The requirement for informed consent was waived considering the retrospective nature of the study. A unified patient data set was used to collect pertinent information, including clinical history, diagnostic workup, and operative and postoperative data from medical records. This report follows the Strengthening the Reporting of Observational Studies in Epidemiology (STROBE) reporting guideline.

### Definitions and End Points

Cardiogenic shock was defined as persistent hypotension (systolic blood pressure <90 mm Hg) with reduction in cardiac index (<1.8 L/min/m^2^) despite maximal treatment. Urgent surgery was defined as surgery required during the same hospitalization for patients who were not admitted in an elective regimen. Emergent surgery was defined as surgery that occurred within 24 hours, while salvage surgery was defined as procedures for patients who required cardiopulmonary resuscitation while going to the operating room. Among surgical techniques, infarct exclusion was described as postinfarction VSR repair accomplished according to the technique described by David et al^11^ or any of its subsequent modifications,^14^ while other techniques included infarct excision and any other technique adopted, comprising sandwich technique and direct closure, with or without the use of a patch.

The primary end point of this study was early mortality, defined as death from any cause within 30 days after surgery or during the same hospitalization related to the operation. Intraoperative mortality was defined as death occurring during the operation. The secondary end point was the identification of prognostic factors associated with early mortality.

### Statistical Analysis

Continuous variables were tested for distribution normality with the Shapiro-Wilk test and reported as mean and SD (for variables not violating the normality assumption) or median and IQR (for variables violating the normality assumption). Categorical variables were reported as frequency and percentage. For patients who survived vs those who died, continuous variables were compared individually with the *t* test or Mann-Whitney *U* test, while categorical variables were compared with the χ^2^ or Fisher exact test, as appropriate. Subsequently, variables of clinical interest that achieved a *P* < .10 at the univariate analysis and had less than 20% missing data were tested for multicollinearity and then entered into a multivariate logistic regression analysis, tested with the Hosmer-Lemeshow goodness-of-fit test, to identify independent variables associated with in-hospital mortality. Data analyses were performed using the software package SPSS Statistics version 26.0 for Windows (IBM). A 2-tailed *P* < .05 was considered statistically significant.

## Results

### Clinical Characteristics

A total of 475 patients were included in this study. Baseline and preoperative patients’ characteristics are presented in Table 1. The mean (SD) age was 68.5 (10.1) years, and 290 (61.1%) were men. The most frequent comorbidity was hypertension, followed by dyslipidemia and diabetes. VSR occurred after ST-elevation myocardial infarction in nearly 90% of patients (366 of 416 [88.0%]) and during the first ischemic episode in three-quarters of patients (348 of 475 [73.3%]), while only 67 (14.1%) had undergone previous revascularization procedures. Preoperative coronary angiography was performed in 397 patients (83.6%), showing multivessel coronary artery disease (CAD) in 226 cases (56.9%). Nearly half of patients developed cardiogenic shock before the operation (213 patients [44.8%]), and approximately 1 in 10 patients (46 [9.7%]) had cardiac arrest. The median (IQR) time from AMI to VSR diagnosis was 2.0 (0.3-5.6) days, while the median (IQR) time from VSR to surgery was 2.0 (0.5-7.0) days. Preoperative intra-aortic balloon pump (IABP) or extracorporeal membrane oxygenation (ECMO) were required in 273 patients (57.5%) and 32 patients (6.7%), respectively. Emergent or salvage operation was performed in nearly half of the patients (212 [44.6%]).

**Table 1.** Baseline and Preoperative Characteristics

Variable	Patients, No. (%)			*P* value
	All (N = 475)	Survived (n = 283)	Died (n = 192)	
Age, mean (SD), y	68.5 (10.1)	66.8 (10.1)	71 (9.7)	<.001
Men	290 (61.1)	180 (63.6)	110 (57.3)	.17
Women	185 (48.9)	103 (36.4)	82 (42.7)	
BMI, mean (SD)	26.1 (4.1)	25.9 (4.1)	26.3 (4.1)	.26
Hypertension	278 (58.5)	171 (60.4)	107 (55.7)	.31
Dyslipidemia	152 (32)	98 (34.6)	54 (28.1)	.14
Diabetes	107 (22.5)	60 (21.2)	47 (24.5)	.40
Stroke or TIA	26 (5.5)	13 (4.6)	13 (6.8)	.31
Smoking habit	177 (37.3)	115 (40.6)	62 (32.3)	.07
COPD	42 (8.8)	22 (7.8)	20 (10.4)	.32
Chronic kidney disease	61 (12.8)	28 (9.9)	33 (17.2)	.02
Peripheral vascular disease	42 (8.8)	29 (10.2)	13 (6.8)	.19
LVEF <45%	205 (43.2)	117 (41.3)	88 (45.8)	.33
Hemodynamic presentation				
Cardiogenic shock	213 (44.8)	95 (33.6)	118 (61.5)	<.001
Cardiac arrest	46 (9.7)	14 (4.9)	32 (16.7)	<.001
Cardiac tamponade	21 (4.4)	11 (3.9)	10 (5.2)	.49
Preoperative IABP	273 (57.5)	149 (52.7)	124 (64.6)	.01
Preoperative ECMO	32 (6.7)	13 (4.6)	19 (9.9)	.02
Preoperative revascularization	158 (33.3)	79 (27.9)	79 (41.1)	.003
Time to surgery ≥7 d^a^	152 (47.9)	105 (57.4)	47 (35.1)	<.001
Surgical status				
Elective	77 (16.2)	55 (19.4)	22 (11.5)	<.001
Urgent	186 (39.2)	124 (43.8)	62 (32.3)	
Emergent or salvage	212 (44.6)	104 (36.8)	108 (56.2)	

Abbreviations: BMI, body mass index (calculated as weight in kilograms divided by height in meters squared); COPD, chronic obstructive pulmonary disease; ECMO, extracorporeal membrane oxygenation; IABP, intra-aortic balloon pump; LVEF, left ventricular ejection fraction; TIA, transient ischemic attack.

^a^ Data available for 317 patients (66.7%).

### Surgical Repair

Operative and perioperative data are reported in Table 2. Anterior/apical VSR was slightly more common. Another concomitant post-AMI mechanical complication (ie, left ventricular free-wall rupture or papillary muscle rupture) was present in 25 patients (5.3%). Concomitant coronary artery bypass graft (CABG) was performed in half of patients, while other concomitant procedures were required in 76 cases (16.0%), mostly including valve procedures or surgical ventricular reconstruction. Moreover, only 72 patients who underwent concomitant CABG (15.2%) also received a preoperative percutaneous revascularization. Nearly two-thirds of patients required postoperative IABP (303 [63.8%]), while ECMO was necessary in approximately 15% of cases (65 [13.7%]). Other MCS adopted included implantation of an Impella device in 5 patients (1.1%) and right or left ventricular assist devices in 2 patients (0.4%) each. Rethoracotomy for bleeding was required in approximately 10% of cases (51 [11.1%]). Residual/recurrent VSR was reported in 59 patients (12.9%), demanding reoperation in 25 (42.4%) of them; in 2 cases (3.4%), residual VSR was closed percutaneously.

**Table 2.** Operative and Perioperative Data

Variable	Patients, No. (%)			*P* value
	All (N = 475)	Survived (n = 283)	Died (n = 192)	
VSR site^a^				
Anterior and apical	235 (53.9)	149 (58.7)	86 (47.3)	.02
Posterior	201 (46.1)	105 (41.3)	96 (52.7)	
Concomitant postinfarction mechanical complications	25 (5.3)	13 (4.6)	12 (6.3)	.43
Repair technique^b^				
Infarct exclusion	78 (17.4)	50 (19.1)	28 (15	.26
Other techniques	371 (82.6)	212 (80.9)	159 (85)	
CPB time, median (IQR), min	137.0 (104.0-181.0)	128.0 (98.0-169.5)	152.5 (118.8-192.5)	.001
ACC time, median (IQR), min	90.0 (69.0-115.0)	85.0 (67.3-112.8)	95.5 (73.3-121.8)	.01
Concomitant CABG	235 (49.5)	144 (50.9)	91 (47.4)	.46
Other concomitant procedures				
FWR or PMR repair	21 (4.4)	12 (4.2)	9 (4.7)	.82
Other procedures	76 (16.0)	48 (17.0)	28 (14.6)	.49
Postoperative inotropes	364 (76.6)	213 (75.3)	151 (78.6)	.39
Postoperative IABP	303 (63.8)	163 (57.6)	140 (72.9)	.001
Postoperative ECMO	65 (13.7)	23 (8.1)	42 (21.9)	<.001
Rethoracotomy for bleeding^c^	51 (11.1)	20 (7.1)	31 (17.6)	<.001
Recurrent or residual VSR^c^	59 (12.9)	33 (11.7)	26 (14.8)	.33
Requiring reoperation	25 (5.4)	15 (5.3)	10 (5.7)	.86
Not requiring reoperation	34 (7.4)	18 (6.4)	16 (9.1)	.28

Abbreviations: ACC, aortic cross-clamp; CABG, coronary artery bypass grafting; CPB, cardiopulmonary bypass; ECMO, extracorporeal membrane oxygenation; FWR, free-wall rupture; IABP, intra-aortic balloon pump; PMR, papillary muscle rupture; VSR, ventricular septal rupture.

^a^ Data available for 436 patients (91.8%).

^b^ Data available for 449 patients (94.5%).

^c^ Among 459 patients who survived surgery.

### Postoperative Outcomes

Early postoperative outcomes are reported in Table 3. The most common complications identified among surgical survivors were low cardiac output syndrome (LCOS) and acute kidney injury, followed by sepsis, atrial fibrillation, and pneumonia.

**Table 3.** Postoperative Outcomes and Causes of In-Hospital Mortality

Variable	Patients, No. (%) (N = 475)
Intraoperative mortality	16 (3.4)
Ventilation time, mean (SD), d	5.2 (8.3)
ICU stay, mean (SD), d	11 (23)
Hospital stay, mean (SD), d^a^	23.4 (27.4)
In-hospital mortality	192 (40.4)
Causes of death^b^	
Intraoperative	16 (8.3)
No CPB weaning	11 (5.7)
Incontrollable bleeding	5 (2.9)
Low cardiac output syndrome	70 (36.5)
Multiorgan failure	53 (27.6)
Recurrent or residual VSR	8 (4.2)
Sepsis	7 (3.6)
Arrhythmia	6 (3.1)
Cerebrovascular accident	6 (3.1)
Acute kidney injury	6 (3.1)
Bowel infarction	6 (3.1)
Free-wall rupture	4 (2.1)
Acute myocardial infarction	3 (1.6)
Pneumonia	3 (1.6)
Unknown	4 (2.1)
Postoperative complications^c^	
Cardiac	
Low cardiac output syndrome	115 (25.1)
Right ventricular failure	29 (6.3)
Free-wall rupture	5 (1.1)
Cardiac tamponade	16 (3.5)
Atrial fibrillation	48 (10.5)
Pacemaker implant	13 (2.8)
Cardiac arrest	16 (3.5)
Acute myocardial infarction	9 (2)
Infectious	
Sepsis	49 (10.7)
Mediastinitis	6 (1.3)
Pulmonary	
Pneumonia	46 (10)
Respiratory distress syndrome	23 (5)
Tracheostomy	9 (2)
Kidney	
Acute kidney injury	93 (20.3)
Kidney replacement therapy	41 (8.9)
Neurological	
Cerebrovascular accident	25 (5.4)
Delirium	22 (4.8)
Other	
Gastrointestinal complications	13 (2.8)
Limb ischemia	10 (2.2)
Coagulopathy	7 (1.5)

Abbreviations: CPB, cardiopulmonary bypass; ICU, intensive care unit; VSR, ventricular septal rupture.

^a^ Among 283 patients who survived to discharge.

^b^ Among 192 patients who died.

^c^ Among 459 patients who survived surgery.

Early mortality occurred in 192 patients (40.4%), with 16 (3.4%) intraoperative deaths, either due to ventricular failure, precluding cardiopulmonary bypass (CPB) weaning, or incontrollable bleeding. LCOS and multiorgan failure were by far the most common causes of early mortality (LCOS, 70 [36.5%]; multiorgan failure, 53 [27.6%]); other cardiac causes accounted for approximately one-tenth of deaths (Table 3). Overall, 8 patients (1.9%) died because of VSR recurrence. A difference in early mortality was observed among different centers (median [IQR] mortality, 41.1% [25.8%-49.5%]). In the nearly 20 years considered for the study, the mortality rate did not change substantially, with a median (IQR) yearly mortality of 41.7% (32.6%-50.0%) (eFigure in the Supplement). Most patients were hospitalized for nearly 1 month after surgical correction, with a few reaching nearly 3 months of hospitalization.

**Figure 1.**
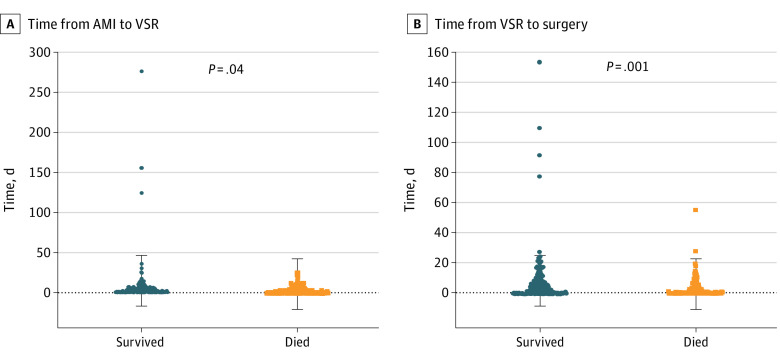
Mean Timing of Ventricular Septal Rupture (VSR) Occurrence and Repair AMI indicates acute myocardial infarction. Dots represent individual cases, and the whiskers mark the SD.

At univariate analysis, older age, smoking, chronic kidney disease, preoperative cardiogenic shock or cardiac arrest, percutaneous revascularization, preoperative need of IABP or ECMO, time from AMI to surgery of less than 7 days, and urgent and emergent/salvage surgery were associated with early mortality (Table 1). For example, cardiogenic shock (survived: 95 [33.6%]; died, 118 [61.5%]; P < .001) and early surgery (time to surgery ≥7 days, survived: 105 [57.4%]; died, 47 [35.1%]; P < .001) were associated with lower survival. Similarly, a significantly higher mortality rate was observed in patients with posterior VSR, longer CPB and cross-clamp times, and in those who required rethoracotomy for bleeding or postoperative support with IABP or ECMO. As presented in Figure 1, patients who died had significantly shorter time from AMI to VSR occurrence compared with those who survived (mean [SD] time, 3.2 [4.1] days vs 8.9 [29.8] days; *P* = .04) and shorter interval from VSR occurrence to surgery (mean [SD] time, 3.3 [6.1] days vs 7.8 [16.1] days; *P* < .001). However, since data from these last 2 variables as well as for the timing of AMI surgery timing were complete in less than 80% of patients, they were not included in the multivariate model.

**Figure 2.**
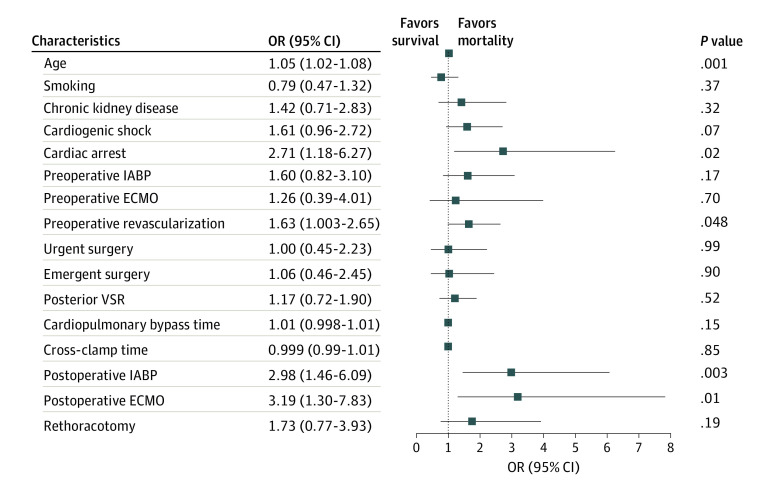
Association of Patient and Clinical Characteristics With Early Mortality ECMO indicates extracorporeal membrane oxygenation; IABP, intra-aortic balloon pump; VSR, ventricular septal rupture.

Multivariate analysis showed that older age (odds ratio [OR], 1.05; 95% CI, 1.02-1.08; *P* = .001), preoperative cardiac arrest (OR, 2.71; 95% CI, 1.18-6.27; *P* = .02), preoperative percutaneous revascularization (OR, 1.63; 95% CI, 1.003-2.65; *P* = .048), and postoperative need of IABP (OR, 2.98; 95% CI, 1.46-6.09; *P* = .003) or ECMO (OR, 3.19; 95% CI, 1.30-7.38; *P* = .01) support were independently associated with early mortality. ORs with 95% CIs are presented in Figure 2.

## Discussion

VSR is a rare but life-threatening mechanical complication of AMI. Thanks to the continuous improvement of early reperfusion strategies, including thrombolysis and percutaneous coronary intervention (PCI), its incidence has decreased from 2% to approximately 0.2% of AMI.^3,4,5,6,18^ If treated conservatively, this condition is almost inevitably fatal.^2^ However, despite prompt intervention, in-hospital mortality still ranges from 19% to 65%.^1,11,12^ Using data from 26 centers around the world, we analyzed the early outcomes of patients undergoing surgical repair of postinfarction VSR during the last 20 years.

The major findings of this study include the following: (1) the early mortality rate was 40.4%; (2) older age, preoperative cardiac arrest and percutaneous revascularization, and postoperative need for IABP or ECMO support were independently associated with early mortality; (3) longer durations from AMI to VSR and from VSR to surgery were associated with lower mortality; and (4) recurrent VSR was not associated with higher mortality. To our knowledge, the current analysis represents the largest multicenter observational study to date evaluating early outcomes among patients undergoing surgical repair of postinfarction VSR.

In our population, older age was independently associated with mortality, in accordance with previous studies.^2,3,16,19^ Although female sex has been historically associated with VSR occurrence and mortality, our sample mostly consisted of men (61.1%); however, we found no association between sex and early mortality.^2,20^ Chronic kidney disease was associated with early mortality, as reported by other studies.^1,14,16,21^

Interestingly, preoperative percutaneous revascularization, including both thrombolysis and PCI, were independently associated with mortality. Although requiring further investigation, this may be related to the higher hemorrhagic risk following percutaneous procedures. Indeed, as previously observed by Becker et al,^20^ thrombolysis may cause myocardial hemorrhage during the lytic state of infarct evolution, thus possibly accelerating VSR occurrence up to the first 24 hours from AMI.^2^ On the other hand, while PCI improves morbidity and mortality in patients with AMI, it has also been shown to reduce the risk of cardiac rupture when compared with thrombolysis.^22^ However even among patients undergoing PCI, myocardial rupture tends to occur earlier than in the prethrombolytic era.^5^ In our study, a shorter time from AMI to VSR occurrence was associated to early mortality (Figure 1A), possibly because of the myocardial hemorrhagic weakening induced by percutaneous revascularization that might also increase the surgical repair difficulty, thereby partially justifying a worse outcome.

However, it should be noted that only one-third of patients underwent preoperative revascularization, given that nearly 20% of patients could not undergo preoperative coronarography, thereby precluding any chance of revascularization, and half of patients received CABG, with limited overlap between percutaneous and surgical revascularization (15.2% of patients). Nevertheless, concomitant CABG was not associated with early mortality, in accordance with some reports.^12,23,24^ The role of concomitant CABG in this setting is still debated because advantages of revascularization are often contrasted by a higher surgical risk and longer CPB and cross-clamp times. Considering that VSR is a post-AMI complication, treatment of the underlying CAD is advocated to provide ischemic border perfusion and protect from the additive long-term ischemic risk of CAD.^25^ Moreover, while most surgeons do not revascularize the infarct-related artery because of technical issues related to the procedure, CABG may be particularly important in multivessel CAD, which has also been associated with higher mortality.^25^

The unfavorable prognosis associated with VSR is strongly influenced by clinical status at time of surgery.^1,6,26^ Indeed, most patients present in labile hemodynamic conditions, ranging from acute pulmonary edema to cardiac arrest.^1,3,16,23^ The negative effect of hemodynamic instability on patients’ survival may also result from other concomitant negative prognostic factors, such as VSR and infarct size, and biventricular impairment.^1,2,24,27,28^ However, in our study, preoperative left ventricular function was not associated with early mortality. On the other hand, nearly half of patients developed cardiogenic shock before surgery, and approximately 10% had cardiac arrest. These conditions were significantly associated with early mortality, in accordance with previous reports.^1,12,14,16,23,26^

The high prevalence of cardiogenic shock often requires aggressive treatment with temporary MCS devices, including IABP.^1,6,29,30^ However, while IABP in this setting is recommended by the current guidelines (Class IIa), ECMO and other MCS devices have not yet been considered.^9^ In our study, both preoperative IABP and ECMO supports were associated with early mortality, in accordance with previous reports, probably due to worse hemodynamic conditions of patients requiring MCS.^1,24^ Nevertheless, considering the number of patients presenting with cardiogenic shock, we observed a low adoption rate of preoperative ECMO, with recent reports ranging from 4% to 22%.^14,16,29^ The use of preoperative MCS devices in VSR represents a relatively recent approach, proposed for patients with more critical illness to improve hemodynamics and delay repair until the patient is in better condition.^6,7,26,29,31,32,33^ Indeed, preoperative MCS adoption is sometimes discouraged by the potential interactions of MCS- and VSR-related pathophysiology, which can be anticipated and carefully managed with a patient-tailored, preimplantation analysis.^7,34,35^

The low adoption of preoperative MCS may also be related to the high proportion of patients undergoing an emergent or salvage procedure (44.6%), probably preferred over attempting to delay the repair until after patient stabilization. However, emergent surgery was associated with a higher mortality, in accordance with most studies.^1,15,16^ Timing of surgery in VSR is still a matter of debate. Indeed, while US guidelines recommend immediate surgery for all patients with VSR, European ones suggest delayed repair in patients who respond well to aggressive therapy.^1,9,10,15,16,28^ Such uncertainty expressed by the guidelines on this topic may itself explain the high rate of early surgery as a general indication for VSR patients in some centers, rather than a forced choice due to unstable hemodynamics. Similar to other reports, we found that the time from VSR occurrence to surgery was significantly shorter for patients who died, reinforcing the association between early intervention and higher mortality and potentially highlighting the need for a paradigm shift in the management of patients with VSR (Figure 1B).^6,15,26,29,30,31,35^ Timing from AMI to VSR and from VSR to surgery were not included in the multivariate analysis because approximately 30% of patients were missing these data; therefore, we could not perform further between-group comparisons. However, it is reasonable to suppose that the evidence provided by such a difference is strong enough to represent a significant result. Despite possible selection biases, usually based on the idea that patients in more stable condition are more likely to survive until a delayed surgery, the improved survival for delayed VSR repair is commonly attributed to the myocardial recovery from ischemia and to the evolution of necrotic, friable tissue into a fibrotic, resistant scar, which is more suitable for repair.^1,6,29,36^

Posterior VSR was associated with higher mortality, in accordance with a meta-analysis published by our group in 2021.^24^ This association may be due to more frequent right ventricular involvement and the more challenging surgical implications of posterior/basal VSR repair.^24,28,37^ Conversely, the presence of other post-AMI mechanical complications or concomitant procedures during surgery was not associated with mortality.

In-hospital mortality was remarkably high (40.4%), although comparable with other reports, confirming that postinfarction VSR is the most lethal cardiac surgical condition, reaching rates as high as 60% of patients.^1,12,16,24^ Moreover, it is noteworthy that 8.3% of deaths were intraoperative, indicating that improvements are needed in the management protocols of these patients.^35^ Indeed, as presented in the eFigure in the Supplement, the temporal trend of early mortality for surgically treated VSR remained high and unchanged over the last 2 decades, despite surgical and technological advancements.^3,24^ Most of the patients died due to LCOS or multiorgan failure, highlighting the severe effects of this condition on biventricular function, despite proper repair.^12^

While the need of rethoracotomy for bleeding was higher among patients who died, residual or recurrent VSR was not associated with higher mortality, even when it required a reintervention, in accordance with other studies.^12,13,37^ However, the VSR recurrence rate remained high (12.9%), and in 8 patients, it also represented the cause of death.

Although compatible with the severity of VSR, the high incidence of LCOS and its systemic effects as either a postoperative complication or cause of death might identify a subgroup with greater clinical severity and/or suboptimal management, especially in the first days following surgery. This may also be reflected by the low adoption rate of more aggressive postoperative MCS devices (13.7% for ECMO and 1.9% for other MCS) besides IABP; these devices may act as a protective measure in the early postoperative period, possibly contributing to improved morbidity and mortality.^7,27,35,36^ In accordance with other reports, our study showed that the postoperative need for IABP or ECMO supports was independently associated with mortality, probably because they were reserved only as last-resort for patients with VSR and more severe clinical conditions.^1,16^ However, broader prophylactic MCS adoption for patients with impaired ventricular function might improve outcomes, as described in various reports, with encouraging results.^7,26,29,35,36^ Nevertheless, further studies are required to examine the potential effects of MCS in both preoperative and postoperative periods on outcomes as well as their impact on overall postinfarct VSR management, including the timing of surgery.

### Limitations

This study has limitations. First, due to its retrospective nature, both selection bias and unmeasured confounders cannot be excluded. Second, the multicenter design required a data collection form with a limited number of variables to avoid missing data; thus, the possibility that nonreported variables could have influenced the results of the analysis cannot be completely ruled out. Moreover, we could not include the timing from AMI to VSR and from VSR to surgery in the multivariate analysis, due to a proportion of missing data approximating 30%. Furthermore, this study was limited to operative outcomes and did not providing information on the durability of surgical repair of post-AMI VSR as well as late outcome. Such outcomes may be also influenced by the small number of patients for each center, which could affect surgical approach uniformity and surgeons’ experience. Additionally, data concerning other VSR patients managed conservatively or who died without surgery in all participating centers are lacking, making a comprehensive analysis of the actual prevalence of VSR not feasible.

## Conclusions

Postinfarction VSR remains a severe condition with a challenging management in all its phases, from preoperative to perioperative periods. Despite many advancements in the management of AMI and in the surgical techniques of VSR repair, early mortality in this study was confirmed to be very high (40.4%), with no signs of improvements over the last 2 decades. Delayed surgery may be associated with better survival. Old age, preoperative cardiac arrest and percutaneous revascularization, and postoperative need for IABP or ECMO were independently associated with early mortality, which was mainly related to LCOS. Further prospective studies addressing enhanced preoperative and perioperative patient management are warranted to hopefully improve the currently suboptimal rate of early mortality.

## Notes

Supplement. **eFigure.** Temporal Trends of In-Hospital Mortality, 2001 to 2019

## References

[1] Arnaoutakis GJ, Zhao Y, George TJ, Sciortino CM, McCarthy PM, Conte JV. Surgical repair of ventricular septal defect after myocardial infarction: outcomes from the Society of Thoracic Surgeons National Database. Ann Thorac Surg. 2012;94(2):436-443. doi:10.1016/j.athoracsur.2012.04.020 22626761 PMC3608099

[2] Crenshaw BS, Granger CB, Birnbaum Y, ; GUSTO-I (Global Utilization of Streptokinase and TPA for Occluded Coronary Arteries) Trial Investigators. Risk factors, angiographic patterns, and outcomes in patients with ventricular septal defect complicating acute myocardial infarction. Circulation. 2000;101(1):27-32. doi:10.1161/01.CIR.101.1.27 10618300

[3] Elbadawi A, Elgendy IY, Mahmoud K, . Temporal trends and outcomes of mechanical complications in patients with acute myocardial infarction. JACC Cardiovasc Interv. 2019;12(18):1825-1836. doi:10.1016/j.jcin.2019.04.039 31537282

[4] Figueras J, Alcalde O, Barrabés JA, . Changes in hospital mortality rates in 425 patients with acute ST-elevation myocardial infarction and cardiac rupture over a 30-year period. Circulation. 2008;118(25):2783-2789. doi:10.1161/CIRCULATIONAHA.108.776690 19064683

[5] French JK, Hellkamp AS, Armstrong PW, . Mechanical complications after percutaneous coronary intervention in ST-elevation myocardial infarction (from APEX-AMI). Am J Cardiol. 2010;105(1):59-63. doi:10.1016/j.amjcard.2009.08.653 20102891

[6] Jones BM, Kapadia SR, Smedira NG, . Ventricular septal rupture complicating acute myocardial infarction: a contemporary review. Eur Heart J. 2014;35(31):2060-2068. doi:10.1093/eurheartj/ehu248 24970335

[7] Gambaro A, Rosenberg A, Galiatsou E, Stock UA. Pros and cons of different types of mechanical circulatory support device in case of postinfarction ventricular septal defect. ASAIO J. 2021;67(6):e110-e113. doi:10.1097/MAT.0000000000001290 33060409

[8] Giblett JP, Jenkins DP, Calvert PA. Transcatheter treatment of postinfarct ventricular septal defects. Heart. 2020;106(12):878-884. doi:10.1136/heartjnl-2019-315751 32111641

[9] Ibanez B, James S, Agewall S, ; ESC Scientific Document Group. 2017 ESC guidelines for the management of acute myocardial infarction in patients presenting with ST-segment elevation: The Task Force for the Management of Acute Myocardial Infarction in Patients Presenting With ST-Segment Elevation of the European Society of Cardiology (ESC). Eur Heart J. 2018;39(2):119-177. doi:10.1093/eurheartj/ehx393 28886621

[10] O’Gara PT, Kushner FG, Ascheim DD, . 2013 ACCF/AHA guideline for the management of ST-elevation myocardial infarction: a report of the American College of Cardiology Foundation/American Heart Association Task Force on Practice Guidelines. J Am Coll Cardiol. 2013;61(4):e78-e140. doi:10.1016/j.jacc.2012.11.019 23256914

[11] David TE, Dale L, Sun Z. Postinfarction ventricular septal rupture: repair by endocardial patch with infarct exclusion. J Thorac Cardiovasc Surg. 1995;110(5):1315-1322. doi:10.1016/S0022-5223(95)70054-4 7475183

[12] Cinq-Mars A, Voisine P, Dagenais F, . Risk factors of mortality after surgical correction of ventricular septal defect following myocardial infarction: retrospective analysis and review of the literature. Int J Cardiol. 2016;206:27-36. doi:10.1016/j.ijcard.2015.12.011 26773765

[13] Malhotra A, Patel K, Sharma P, . Techniques, timing and prognosis of post infarct ventricular septal repair: a re-look at old dogmas. Braz J Cardiovasc Surg. 2017;32(3):147-155. doi:10.21470/1678-9741-2016-0032 28832791 PMC5570397

[14] Okamoto Y, Yamamoto K, Asami F, . Early and midterm outcomes of triple patch technique for postinfarction ventricular septal defects. J Thorac Cardiovasc Surg. 2016;151(6):1711-1716. doi:10.1016/j.jtcvs.2016.01.056 27045043

[15] Mantovani V, Mariscalco G, Leva C, Blanzola C, Sala A. Surgical repair of post-infarction ventricular septal defect: 19 years of experience. Int J Cardiol. 2006;108(2):202-206. doi:10.1016/j.ijcard.2005.05.007 15950300

[16] Sakaguchi G, Miyata H, Motomura N, . Surgical repair of post-infarction ventricular septal defect—findings from a Japanese national database. Circ J. 2019;83(11):2229-2235. doi:10.1253/circj.CJ-19-0593 31511450

[17] World Medical Association. World Medical Association Declaration of Helsinki: ethical principles for medical research involving human subjects. JAMA. 2013;310(20):2191-2194. doi:10.1001/jama.2013.281053. 24141714

[18] Moore CA, Nygaard TW, Kaiser DL, Cooper AA, Gibson RS. Postinfarction ventricular septal rupture: the importance of location of infarction and right ventricular function in determining survival. Circulation. 1986;74(1):45-55. doi:10.1161/01.CIR.74.1.45 3708777

[19] Pojar M, Harrer J, Omran N, Turek Z, Striteska J, Vojacek J. Surgical treatment of postinfarction ventricular septal defect: risk factors and outcome analysis. Interact Cardiovasc Thorac Surg. 2018;26(1):41-46. doi:10.1093/icvts/ivx230 29049690

[20] Becker RC, Gore JM, Lambrew C, . A composite view of cardiac rupture in the United States National Registry of Myocardial Infarction. J Am Coll Cardiol. 1996;27(6):1321-1326. doi:10.1016/0735-1097(96)00008-3 8626938

[21] Moreyra AE, Huang MS, Wilson AC, Deng Y, Cosgrove NM, Kostis JB; MIDAS Study Group (MIDAS 13). Trends in incidence and mortality rates of ventricular septal rupture during acute myocardial infarction. Am J Cardiol. 2010;106(8):1095-1100. doi:10.1016/j.amjcard.2010.06.013 20920645

[22] López-Sendón J, Gurfinkel EP, Lopez de Sa E, ; Global Registry of Acute Coronary Events (GRACE) Investigators. Factors related to heart rupture in acute coronary syndromes in the Global Registry of Acute Coronary Events. Eur Heart J. 2010;31(12):1449-1456. doi:10.1093/eurheartj/ehq061 20231153

[23] Khan MY, Waqar T, Qaisrani PG, . Surgical repair of post-infarction ventricular septal rupture: determinants of operative mortality and survival outcome analysis. Pak J Med Sci. 2018;34(1):20-26. doi:10.12669/pjms.341.13906 29643872 PMC5857013

[24] Matteucci M, Ronco D, Corazzari C, . Surgical repair of postinfarction ventricular septal rupture: systematic review and meta-analysis. Ann Thorac Surg. 2021;112(1):326-337. doi:10.1016/j.athoracsur.2020.08.050 33157063

[25] Barker TA, Ramnarine IR, Woo EB, . Repair of post-infarct ventricular septal defect with or without coronary artery bypass grafting in the northwest of England: a 5-year multi-institutional experience. Eur J Cardiothorac Surg. 2003;24(6):940-946. doi:10.1016/S1010-7940(03)00465-2 14643812

[26] Hobbs R, Korutla V, Suzuki Y, Acker M, Vallabhajosyula P. Mechanical circulatory support as a bridge to definitive surgical repair after post-myocardial infarct ventricular septal defect. J Card Surg. 2015;30(6):535-540. doi:10.1111/jocs.12561 25940559

[27] Jacob S, Patel MJ, Lima B, . Using extracorporeal membrane oxygenation support preoperatively and postoperatively as a successful bridge to recovery in a patient with a large infarct-induced ventricular septal defect. Proc (Bayl Univ Med Cent). 2016;29(3):301-304. doi:10.1080/08998280.2016.11929443 27365878 PMC4900776

[28] Coskun KO, Coskun ST, Popov AF, . Experiences with surgical treatment of ventricle septal defect as a post infarction complication. J Cardiothorac Surg. 2009;4(3):3. doi:10.1186/1749-8090-4-3 19126196 PMC2631454

[29] Rob D, Špunda R, Lindner J, . A rationale for early extracorporeal membrane oxygenation in patients with postinfarction ventricular septal rupture complicated by cardiogenic shock. Eur J Heart Fail. 2017;19(suppl 2):97-103. doi:10.1002/ejhf.852 28470920

[30] Ariza-Solé A, Sánchez-Salado JC, Sbraga F, . The role of perioperative cardiorespiratory support in post infarction ventricular septal rupture-related cardiogenic shock. Eur Heart J Acute Cardiovasc Care. 2020;9(2):128-137. doi:10.1177/2048872618817485 30525871

[31] Watkins AC, Maassel NL, Ghoreishi M, . Preoperative venoarterial extracorporeal membrane oxygenation slashes risk score in advanced structural heart disease. Ann Thorac Surg. 2018;106(6):1709-1715. doi:10.1016/j.athoracsur.2018.07.038 30236527

[32] Morimura H, Tabata M. Delayed surgery after mechanical circulatory support for ventricular septal rupture with cardiogenic shock. Interact Cardiovasc Thorac Surg. 2020;31(6):868-873. doi:10.1093/icvts/ivaa185 33118011

[33] Matteucci M, Fina D, Jiritano F, . The use of extracorporeal membrane oxygenation in the setting of postinfarction mechanical complications: outcome analysis of the Extracorporeal Life Support Organization Registry. Interact Cardiovasc Thorac Surg. 2020;31(3):369-374. doi:10.1093/icvts/ivaa108 32728712

[34] Pahuja M, Schrage B, Westermann D, Basir MB, Garan AR, Burkhoff D. Hemodynamic effects of mechanical circulatory support devices in ventricular septal defect: results from a computer simulation model. Circ Heart Failure. 2019;12(7):e005981. doi:10.1161/CIRCHEARTFAILURE.119.005981 31296094

[35] Ronco D, Matteucci M, Ravaux JM, . Mechanical circulatory support as a bridge to definitive treatment in post-infarction ventricular septal rupture. JACC Cardiovasc Interv. 2021;14(10):1053-1066. doi:10.1016/j.jcin.2021.02.046 34016403

[36] Gregoric ID, Kar B, Mesar T, . Perioperative use of TandemHeart percutaneous ventricular assist device in surgical repair of postinfarction ventricular septal defect. ASAIO J. 2014;60(5):529-532. doi:10.1097/MAT.0000000000000108 25010911

[37] Jeppsson A, Liden H, Johnsson P, Hartford M, Rådegran K. Surgical repair of post infarction ventricular septal defects: a national experience. Eur J Cardiothorac Surg. 2005;27(2):216-221. doi:10.1016/j.ejcts.2004.10.037 15691673

