# Supplementary material for: Surgical Treatment of Postinfarction Ventricular Septal Rupture
Source: JAMA Netw Open. 2021 Oct 20;4(10):e2128309. doi: 10.1001/jamanetworkopen.2021.28309 (PMC8529403; doi:10.1001/jamanetworkopen.2021.28309)
Supplement: Supplement. — eFigure. Temporal Trends of In-Hospital Mortality, 2001 to 2019 [file jamanetwopen-e2128309-s001.pdf]

Supplemental Online Content

Ronco D, Matteucci M, Kowalewski M, et al. Surgical treatment of postinfarction ventricular septal rupture. *JAMA Netw Open.* 2021;4(10):e2128309.
doi:10.1001/jamanetworkopen.2021.28309

eFigure. Temporal Trends of In-Hospital Mortality, 2001 to 2019

This supplemental material has been provided by the authors to give readers additional information about their work.

© 2021 Ronco D et al. *JAMA Network Open.*

eFigure. Temporal Trends of In-Hospital Mortality, 2001 to 2019

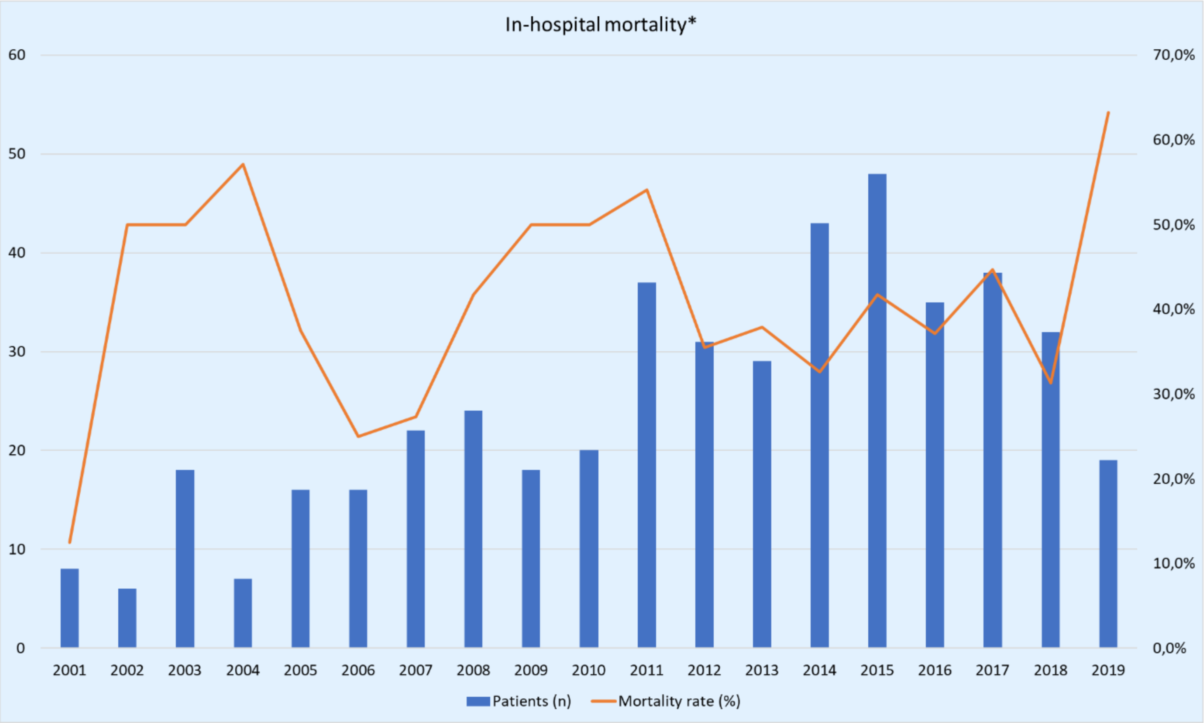

*Year of surgery available in 467 patients (98.3%).

© 2021 Ronco D et al. *JAMA Network Open.*
